# Microfluidic Study of the Effect of Nanosuspensions on Enhanced Oil Recovery

**DOI:** 10.3390/nano12030520

**Published:** 2022-02-02

**Authors:** Maxim I. Pryazhnikov, Andrey V. Minakov, Andrey I. Pryazhnikov, Ivan A. Denisov, Anton S. Yakimov

**Affiliations:** 1 Department of Science and Innovation, Siberian Federal University, 660041 Krasnoyarsk, Russia; mpryazhnikov@sfu-kras.ru (M.I.P.); apryazhnikov@sfu-kras.ru (A.I.P.); idenisov@sfu-kras.ru (I.A.D.); asyakimov@gmail.com (A.S.Y.); 2 Laboratory of Heat Exchange Control in Phase and Chemical Transformations, Kutateladze Institute of Thermophysics, 630090 Novosibirsk, Russia

**Keywords:** SiO_2_-based nanosuspension, enhanced oil recovery, microfluidics, nanoparticle concentration

## Abstract

The essential advantages of microfluidic studies are the excellent visualization of the processes of oil displacement from the porous medium model, simple cleaning, and the possibility of the repeated use of the microfluidic chip. The present article deals with the process of oil displacement by suspension flooding using a microfluidic chip, simulating a porous medium, and the suspensions of silicon dioxide nanoparticles (22 nm). The mass concentration of nanoparticles in suspensions ranged from 0.1 to 2 wt%. Five mass concentrations (0.125 wt%, 0.25 wt%, 0.5 wt%, 1 wt% and 2 wt%) were considered. The article presents the experimental photographs of the oil displacement process by water and SiO_2_ suspension. It is shown that, with the increasing concentration of nanoparticles, the oil recovery factor increases. A significant effect is observed at 0.5 wt% concentration of nanoparticles. It is shown that the increase in oil recovery during flooding by SiO_2_ suspension with the maximum concentration was 16%.

## 1. Introduction

Currently, less than half of the initial oil is extracted during the main production [1,2]. A slight improvement in oil recovery will lead to a colossal amount of additional extracted oil on the scale of fields. Oil extraction by the primary method is carried out due to natural forces (intrastratal pressure, fluid expansion, compressibility of the rock). During the well operation, the intrastratal pressure decreases. At some point, the underground pressure becomes insufficient for further oil production. In this case, a set of measures to enhanced oil recovery is used. These measures include secondary methods [3,4,5], designed to increase intrastratal pressure, and tertiary methods [6,7,8], associated with increased oil mobilization.

The traditional way to study enhanced oil recovery is by studying core flooding [9,10,11,12]. Core flooding provides some information about the kinetics and amount of extracted oil. However, it has several disadvantages; for example, the complexity and duration of testing, the lack of observation of the mechanism, and phenomena occurring at the microlevel of pores, as well as the reproducibility of the results. As known, most of the oil is retained by capillary forces in the pores. The volume of oil remaining in the reservoir is determined by the ratio of inertia forces, displacing the oil and capillary forces retaining it [13].

Microfluidic studies using rock micromodels are becoming an alternative to core flooding [14,15,16,17,18,19,20,21,22]. The development of microfluidics is associated with a significant reduction in the size of devices and the improvement of their technical characteristics. Modern microfluidic models of a porous medium, in which the characteristic channel size ranges from several microns to hundreds of microns, are actively used in studies to enhance oil recovery [23,24,25,26,27,28,29,30]. The relevance of recent research is related to the creation and selection of the composition of displacing fluids to increase oil recovery during flooding, as well as the study of the permeability of a microporous medium. The advantages of microfluidic research in comparison with conventional flooding methods are the excellent visualization of the oil displacement processes from the porous medium model, simple cleaning, and the possibility of the repeated use of the microfluidic chip, as well as a wide range of temperatures and pressures in experiments, and excellent chemical compatibility.

The number of studies devoted to the use of microfluidic technologies in relation to the problems of enhancing oil recovery is growing contentiously. Osei-Bonsu et al. [14] used a microporous model, printed using a 3D printer, to study the displacement of oil during flooding with foam. The authors showed how the foam quality affects the flow pattern in a porous medium. Saadat et al. [15,16] conducted a systematic study of the process of oil displacement from a microporous model using salt solutions and surfactants. They examined the effect of the oil properties, the composition of salt solutions and surfactants, and the wettability of the micromodel on the increase in the oil recovery factor (RF). Using low salinity solutions is effective for a water-wetted system, whereas it is better to use surfactants for an oil-wetted one. The authors reported an increase in the oil recovery factor to 19.5% compared to using the brine solution. In [18], it is noted that the increase in the oil recovery factor due to the infiltration of surfactants into oil reached 14% during the flooding of surfactants. Nguyen et al. [19] evaluated the effectiveness of using nitrogen and supercritical carbon monoxide to displace oil. A significant increment of the oil recovery factor was obtained due to the dissolution of carbon monoxide in oil.

One of the most potential applications is the addition of nanoparticles, whose size does not exceed 100 nm, to the displacing fluid, to improve the oil recovery factor. Aadland et al. [17] presented the results of a study of cellulose nanocrystals as a chemical additive to increase oil recovery. Adding cellulose nanoparticles gave 5.8% of the original oil in place, compared with low salinity water. Nilsson et al. [21] studied the effect of the displacing fluid rheology on the fraction of remaining oil in a porous medium. A displacing rheological fluid based on water and high molecular weight polyethylene oxide with SiO_2_ nanoparticles (12 nm) increased the oil recovery factor to 95%, compared to water (45%). Betancur et al. [23] presented the results of the effect of a mixture of surfactants and magnetic iron core-carbon shell of nanoparticles (60 nm) during microfluidic flooding. The oil recovery factor was up to 84%. Rueda et al. [31] showed a beneficial and negative effect on the oil recovery factor when adding SiO_2_ nanoparticles to the Xanthan gum and Scleroglucan biopolymers, respectively. The article [32] presents an analysis of the effect of the pore size distribution on the flow in the carbonate rock. It was shown that using SiO_2_ nanoparticle solutions with anionic surfactant improves oil recovery factor from a core with an inhomogeneous pore size distribution by 8.6%. Alcorn et al. [33] stabilized CO_2_ foams by surface-modified spherical silica nanoparticles. Experiments have shown an increase in the apparent viscosity. The authors note that an increase in apparent viscosity reduces the mobility of CO_2_, which, in turn, can increase the efficiency of CO_2_-enhanced oil recovery.

In recent works, suspensions of nanoparticles (nanofluids) have been proposed as promising displacement fluids during waterflooding in order to increase oil recovery [33,34]. A nanofluid is a suspension (usually water-based) with the addition of nanoparticles of various compositions. Their size and concentration can significantly affect the physical properties of the nanosuspension. Nanofluids are now used in a great number of different applications. An example of the use of nanosuspensions for enhanced oil recovery is given in [33]. The authors of [33] prepared nanofluids based on water and surfactants with amphiphilic Janus-SiO_2_ nanoparticles. They found that oil recovery can be increased by 10% at a nanoparticle concentration of 0.15 wt%. This is because the amphiphilic Janus-SiO_2_ can increase the sweep efficiency of the surfactants and reduce the interfacial tension of the oil–water emulsion, thereby enhancing oil recovery with water flooding. In another work [34], a systematic study of various properties of water-based SiO_2_-nanofluid on the efficiency of oil displacement was carried out using numerical simulations. The effects of nanoparticle concentration and size, displacement fluid flow rate, oil viscosity and core permeability were considered. The addition of nanoparticles to the displacing fluid significantly affects the process of oil displacement from the porous rock. When the mass fraction of nanoparticles increases to 0.5%, the oil recovery can be increased by about 19% compared with water flooding.

A brief review of microfluidic studies on enhanced oil recovery using microfluidic experiments has shown that, to date, sufficient attention has been paid to studies on flooding using emulsions [18], surfactants [26], foams [14], and salt solutions [15,16], as well as microbial-enhanced oil recovery [30], polymer-enhanced oil recovery [21,31], and CO_2_-enhanced oil recovery [19,35]. However, very few works are devoted to the study of oil recovery using nanosuspensions. The current article presents the results of an experimental study of the oil displacement from a microporous medium by SiO_2_-based nanosuspensions. The study was conducted using lab-on-a-chip microfluidic technology. The article shows oil and suspensions flow patterns during flooding, as well as the effect of the SiO_2_ nanoparticle concentration on enhancing oil recovery.

## 2. Materials and Methods

### 2.1. Microfluidic Chip

**Figure 1:**
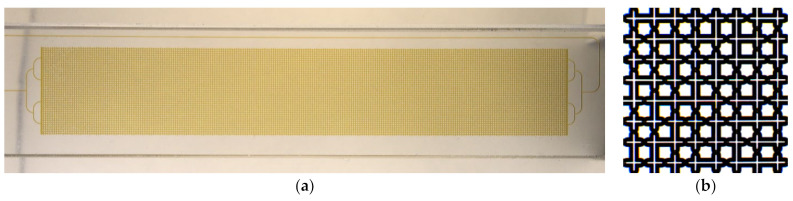
Microfluidic chip (**a**) and its enlarged fragment (**b**).

A microfluidic chip (Dolomite: 3200284) was used in the work, which allows modeling the complex porous structure of rock with regard to research problems in the oil production field (Figure 1). The microfluidic chip was made by etching sodium-lime glass (B270 material) with hydrogen fluoride, followed by thermal bonding. The size of the 4 mm thick microfluidic chip was 92.5 × 15.0 mm^2^. The porous area of the chip had a size of 10 × 60 mm^2^. The chip had one input and one output. The microfluidic chip was connected by a 4-sided upper interface (4 mm) (Dolomite: 3000109) and a linear 4-pin connector (Dolomite: 3000024).

The porous area is formed by repeating (150 times) a 2 × 2 mm^2^ square (Figure 1b). The arrangement of channels in a square represents a grid of 8 × 8 channels, which have an almost circular cross-section (the channel depth and width are 100 and 110 µm, respectively). The channels in the grid have constrictions or “pores” that are randomly distributed to imitate the natural structure of the rock. The grid contains 38 pores with Ø63 µm, 40 pores with Ø85 µm, and 50 straight channels. Additional parameters are presented in Table 1.

### 2.2. Crude Oil and Displacing Fluids

**Figure 2:**
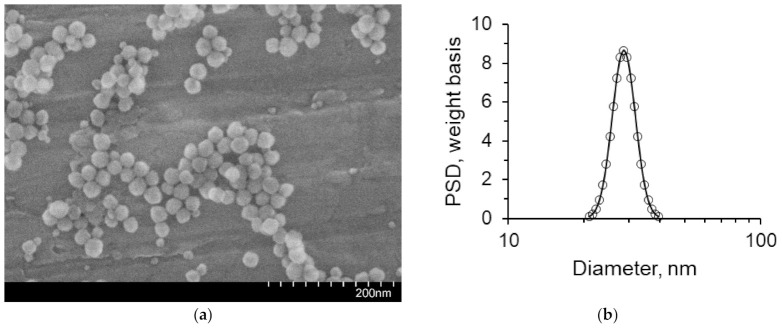
Characterization of the SiO_2_ nanoparticle size: (**a**) SEM photo of primary particles; (**b**) Particle size distribution in a fluid.

Crude oil with a density of 851 kg/m^3^, and a viscosity of 24.6 mPa s, having a temperature of 25 °C, was used in the work. Distilled water and a suspension of silicon dioxide nanoparticles were used as displacing fluids. The mass concentrations of considered nanoparticles ranged from 0.1 to 2 wt%. The concentrations were changed by diluting the Ludox TM-50 (Sigma-Aldrich, St. Louis, MA, USA) suspension with distilled water. The nanoparticles have a spherical shape, with the size of the primary particles being equal to 22 nm. The image of SiO_2_ nanoparticles, obtained by an ultra-high resolution scanning electron microscope (Hitachi S-5500, Tokyo, Japan), is shown in Figure 2a. The particle size distribution in the fluid was obtained using a DT1202 acoustic and electroacoustic spectrometer (Dispersion Technology, Bedford Hills, NY, USA) (Figure 2b). The average hydrodynamic particle size is 30 nm. Suspensions of silicon oxide nanoparticles have high stability (the zeta potential for 1 wt% is −31 mV). The stable dispersion time of the suspensions was two months.

The obtained dependences of the suspension viscosity coefficients on the nanoparticle concentration are well described by the expression [36]:

(1) $$\mu_{\mathrm{susp}}\left(w\right)=\mu_{f}\left(1+aw+bw^{2}\right),$$

where $\mu_{f}$ is the coefficient, corresponding to the water viscosity at 25 °C [mPa s^−1^]; a=2.7 and b=3.6 are constants. Here, the concentration is expressed in mass fractions.

The density of SiO_2_ suspensions $\rho_{\mathrm{susp}}$ was estimated according to [37]:

(2) $$\rho_{\mathrm{susp}}=\rho_{p}\phi +\rho_{f}(1-\phi ),$$

where $\rho_{p}$, $\rho_{f}$ are the densities of particles and fluids, respectively: $\rho_{p}=2.2$ g cm^−3^ and $\rho_{f}=0.997$ g cm^−3^; ϕ is the volumetric fraction of particles in the suspension.

The volumetric fraction ϕ is expressed through the mass fraction by the following relation:

(3) $$\phi =\frac{\rho_{f}w}{\rho_{f}w+\rho_{p}(1-w)},$$

The properties (density and viscosity) of used fluids are presented in Table 2.

### 2.3. Experimental Setup

**Figure 3:**
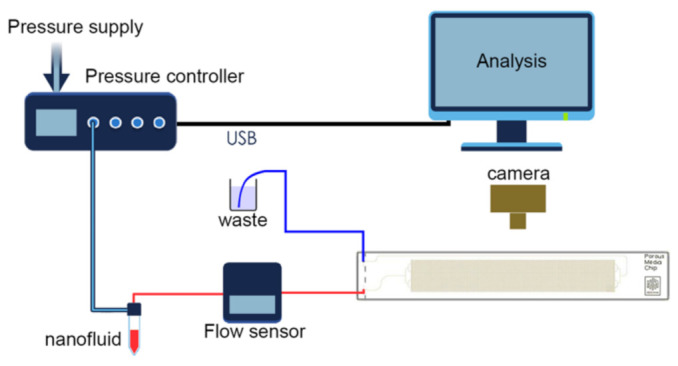
The schematic diagram of the experimental setup.

The schematic diagram of the experimental setup is shown in Figure 3. The displacement fluid flow was controlled using Elveflow OB1 MK3+ multi-channel high-performance microfluidic pressure controller. The controller was equipped with two pressure channels from 0 to 2000 mbar, and from 0 to 8000 mbar. For the 8-bar channel, the accuracy of maintaining pressure was 100 Pa; the response time and pressure setting time were up to 9 and 35 ms, respectively, and the minimum pressure increment was 24 Pa. The flow rate and pressure drop were monitored and controlled using the ESI Microfluidic Software from Elveflow.

An external pressure source (compressor) is required for the operation of the controller. Compressed air from the pressure controller entered a sealed tank with the test displacing fluid. The microfluidic chip was connected to the tank by a 1/16” OD PTFE tube. The microfluidic chip was positioned horizontally on the slide. A Sony RX100IV high-speed camera was located on top of the chip, and a light source was located below for better visualization of the flow pattern.

The MFS3 flow sensor was used, operating within the range from 2.4 to 80 µL min^−1^ with an accuracy of ±5% of the measured value. Sensor response time was up to 70 ms. Calibration of the flow sensor was performed by comparing the set flow rate in the pressure controller of the fluid (water), flown out during a given time interval with the flow rate calculated using the mass and density of the flown fluid. The correction factor was defined as the ratio of a given flow rate to the flow rate measured by weights.

### 2.4. Experimental Procedure

The empty microfluidic chip was initially filled completely with oil, and then the oil was displaced by the flooding of a displacing fluid at a fixed flow rate. At that, 2–3 pore volumes were pumped over. The flooding process was recorded by a high-speed camera, which stored the video for further analysis. After each experiment, the microfluidic chip was thoroughly washed sequentially with dichloromethane, isopropanol, distilled water, and air, and then kept at a temperature of 100 °C.

### 2.5. Estimation of the Oil Recovery Factor

An application has been developed to estimate the oil recovery factor based on obtained photographs of the distribution of oil and the displacing fluid in the pore space of the microfluidic chip. A set of images was obtained by converting video recordings using the free FFmpeg library. To develop the application, the BlackBox Component Builder framework (Oberon microsystems, Zürich, Switzerland) and the FreeImage library were employed. The HSV color model (hue–saturation–value) was used when analyzing the proportion of oil in a microfluidic chip. The application allows one to open the image, select the area of the microfluidic chip, and calculate the oil-bearing capacity by the threshold saturation of pixels.

The oil recovery factor (ORF) was determined as follows:

(4) $$\mathrm{ORF}=1-\frac{N_{\mathrm{oil}}}{N_{\mathrm{PV}}},$$

where $N_{\mathrm{PV}}$ is the number of pixels corresponding to the oil of a completely filled microfluidic chip, $N_{\mathrm{oil}}$ is the number of pixels corresponding to the oil in the course of displacement.

## 3. Results and Discussion

**Figure 4:**
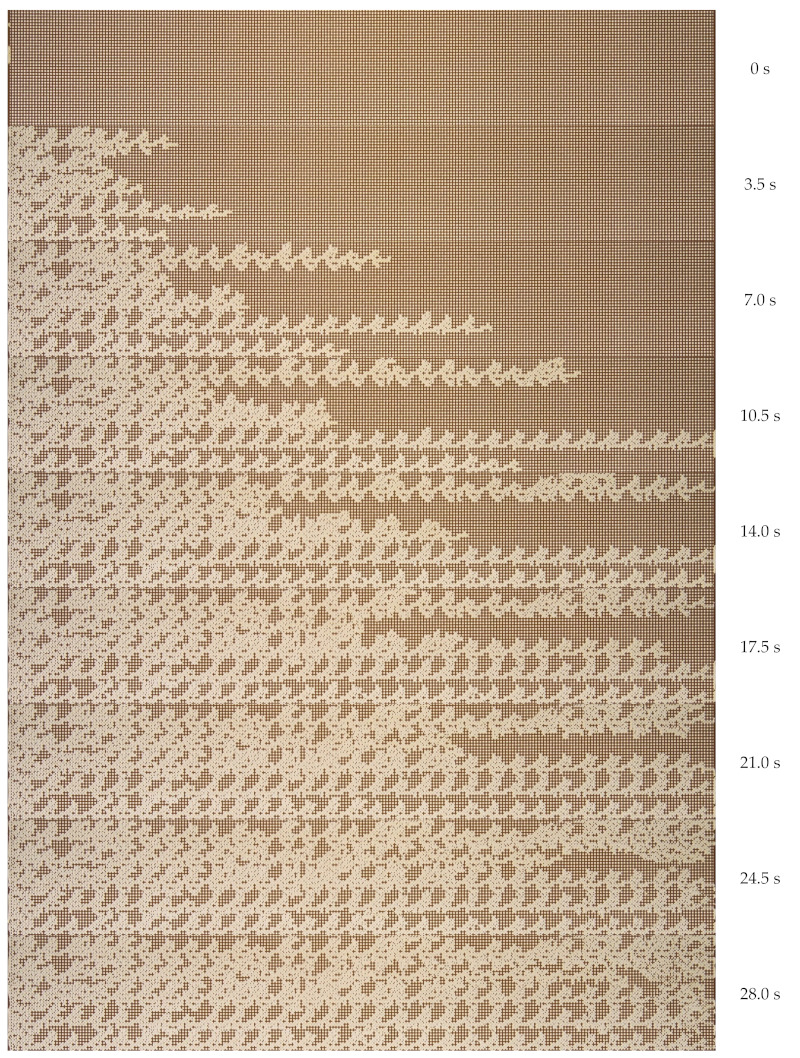
Photos of the oil displacement process from the microfluidic chip by water.

Initially, experimental microfluidic flooding was carried out using water. A microfluidic chip, filled completely with oil, was supplied with water at a volumetric flow rate of 45 µL min^−1^. The dynamic pattern of the oil displacement process by water is shown in Figure 4. The nature of the immiscible liquid–liquid flow during displacement through a porous medium is determined by the capillary number (the ratio of viscous and capillary forces). In this case, viscous forces prevailed over capillary forces. The capillary number was $1.6\times 10^{-3}$. Since the viscosity of water is lower than the viscosity of the displaced oil, the interface in the displacement process is unstable. The moving water flow front has a shape of so-called viscous fingers [38]. Over time, the vicious fingers grow in length. Such fingers quickly break through to the channel exit. The water flows through separate channels. Further water flow occurs through separate channels. At that, approximately 40% of the initial volume of oil remains in a porous medium. After the main viscous fingers have reached the channel exit, the flow process becomes stationary. Further flooding of the microfluidic chip does not lead to an increase in the oil recovery factor. The last pictures of Figure 4 show the steady distribution of oil saturation.

The pressure drop in the microfluidic chip was recorded along with a video recording of the displacement process. The change in the pressure drop over time provided useful information about the beginning of the flooding process and the breakthrough of the displacing fluid from the microfluidic chip.

**Figure 5:**
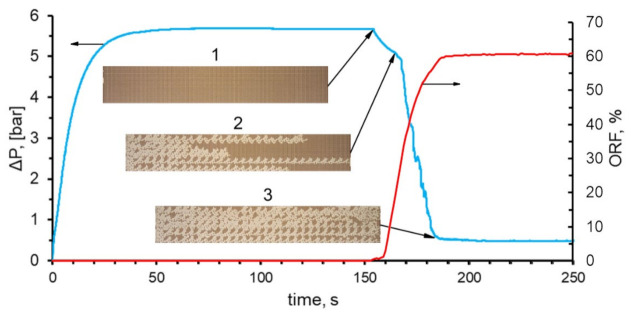
Pressure drop and oil recovery factor in the microfluidic chip during displacement. 1—beginning of the displacement process; 2—the breakthrough of the displacing fluid; 3—displacement process establishment.

A joint analysis of the pressure change and oil recovery factor in the process of flooding with water is shown in Figure 5. The dynamic pattern of the pressure drop is as follows. The pressure drop in the microfluidic chip increases from the moment of turning on the pump, pumping the displacing fluid. At the steady flow of the displacing fluid through the supply pipes, the pressure stabilizes and reaches the maximum value. After the displacing fluid enters the microporous model, the pressure begins to slowly decrease because the viscosity of the displacing fluid is lower than the viscosity of the oil. At that moment, when the displacing fluid reaches the micromodel outlet, the pressure begins to decrease sharply, tending to the atmospheric pressure.

**Figure 6:**
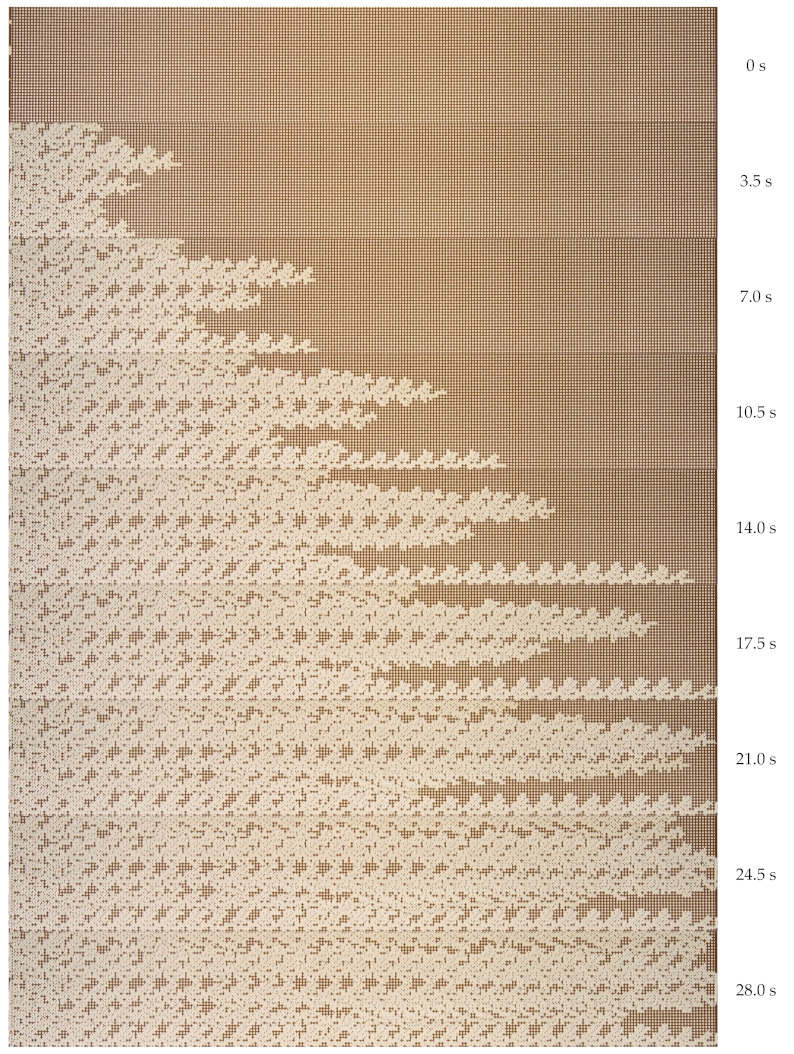
Photos of the oil displacement process from the microfluidic chip by 2 wt% SiO_2_ suspension.

**Figure 7:**
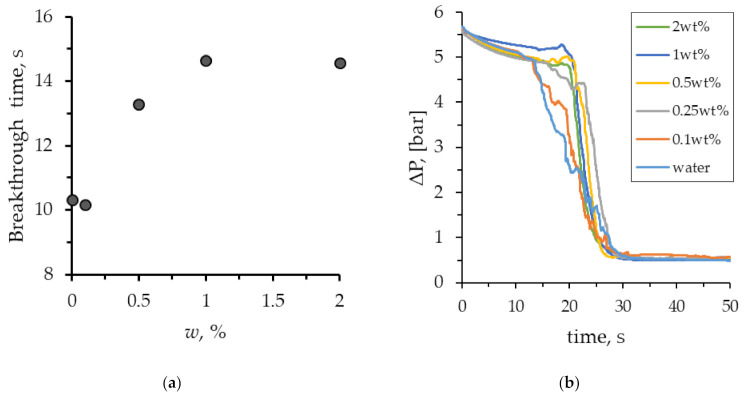
(**a**) The breakthrough time of the suspensions, depending on the concentration of nanoparticles; (**b**) Pressure drop in the microfluidic chip during oil displacement.

Figure 6 shows the oil displacement process by SiO_2_ suspension, with the maximum considered concentration of nanoparticles being equal to 2 wt%. When flooding the micromodel with nanosuspension, the displacement process changes. As can be seen from Figure 6, the movement of the SiO_2_ suspension also occurs in the form of separate jets, but the width of these jets increases significantly. At that, the displacement front becomes significantly more homogeneous. As a result, the breakthrough of the displacing fluid to the exit from the microporous medium occurs significantly later. The dependence of the breakthrough time on the concentration of nanoparticles is shown in Figure 7a. As can be seen, the breakthrough time increases significantly with an increase in the concentration of nanoparticles. Thus, for 2 wt% of nanoparticles, the breakthrough time increases by about 1.5 times. Similar trends were obtained using numerical simulation [34]. This means that at a fixed injection flow rate, the volume of the porous medium, filled with SiO_2_ suspension, increases by about the same rate. The viscous fingers increase in width, covering a larger area. This, certainly, leads to a significant enhancement in oil recovery. This is clearly seen from the comparison of the final Figure 4 and Figure 6.

Figure 7b presents graphs showing the pressure drop behavior when pumping the SiO_2_ suspension through a microchip during the displacement. At that, SiO_2_ nanoparticles influence the dynamic pattern of the pressure drop in the micromodel during flooding. This is due to several factors. The main one is that, with an increase in the concentration of nanoparticles, the time of fluid breakthrough through a porous medium increases. Because of this, the high pressure during the SiO_2_ suspension flow is maintained longer than that for water. An increase in the nanoparticle concentration increased the breakthrough time of the SiO_2_ suspension from the micromodel, as well as led to a sharper decrease in pressure. At a steady flow, after the breakthrough, the values of pressure losses during pumping of water and SiO_2_ suspension were almost the same. This is not surprising, since when adding 2 wt% of silicon oxide nanoparticles, the viscosity of the suspension increased by just 5% compared to pure water. Thus, it was shown that when oil is displaced from a microfluidic chip, pressure losses are weakly dependent on the concentration of nanoparticles. This is very important for the practical application of nanoparticles.

**Figure 8:**
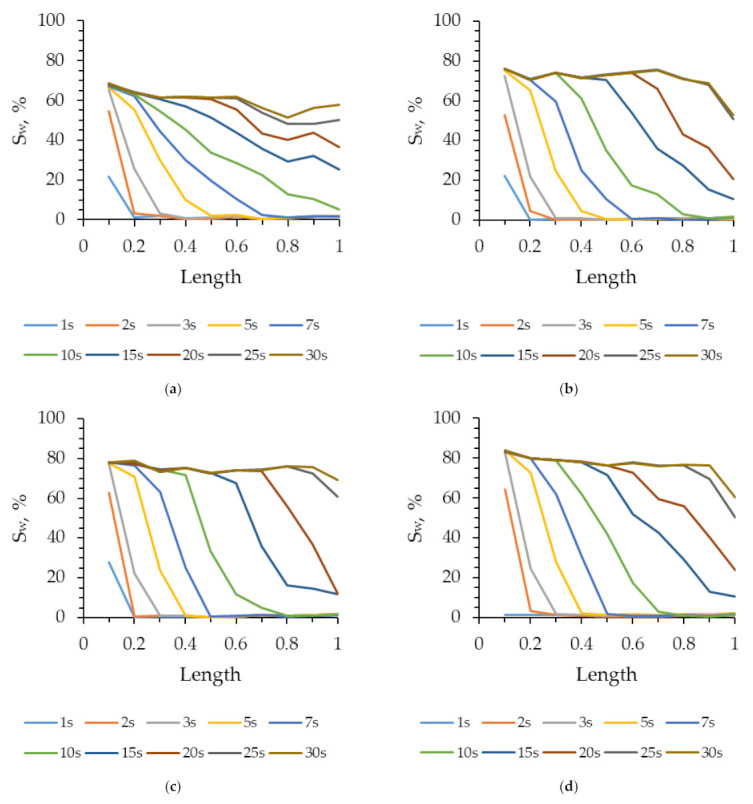
Saturation distribution of water (**a**), 0.5 wt% (**b**), 1 wt% (**c**), and 2 wt% of SiO_2_ suspension (**d**) over the normalized length of the microfluidic chip at different time points.

For a detailed analysis of the displacement process in the microfluidic chip, the latter was divided into 10 equal regions along the length of the microporous medium. The saturation of the displacing fluid ($S_{w}$) was determined in each of these regions. Figure 8 shows the saturation distributions of water and 2 wt% of the SiO_2_ suspension along the length of the microfluidic channel (normalized by the length of the microporous medium) at various points in time. It can be seen from Figure 8 that, firstly, saturation during flooding with water and suspension at the beginning of the microfluidic chip quickly reach a plateau and do not change further. Secondly, the saturation of water in the microfluidic chip is distributed more evenly along the length of the microfluidic chip than that of 2 wt% SiO_2_ suspension. This can be seen from the slope of the saturation distribution curves of the displacing fluid at the same time points, shown in Figure 8. Thirdly, in each subsequent region of the micromodel, the water saturation reaches a steady maximum value more slowly. At that, the maximum steady-state saturation of water at the initial part of the chip was 67%, while the saturation of 2 wt% of SiO_2_ suspension reached 83%. Moreover, it should be noted that the further that the considered area is from the input, the lower the established water saturation level of the displacing fluid is.

**Figure 9:**
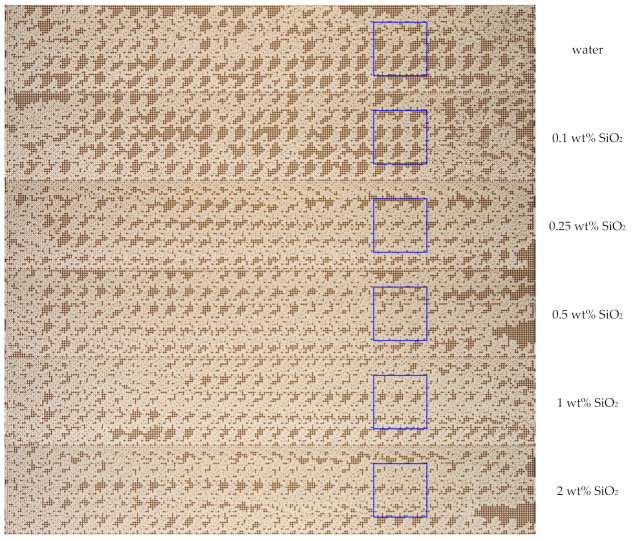
The final distribution pattern of oil and SiO_2_ suspension with different concentrations of nanoparticles in a microfluidic chip.

**Figure 10:**
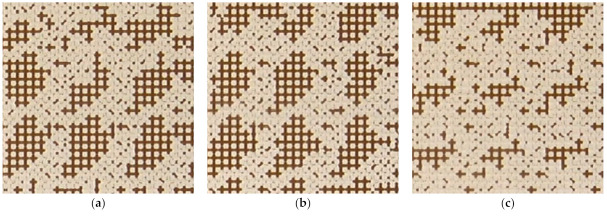

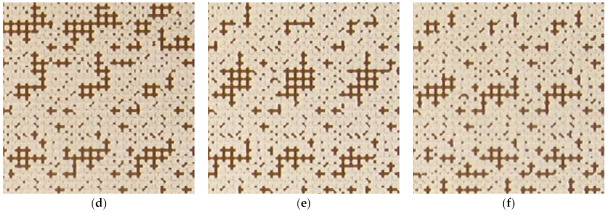
The effect of the nanoparticle concentration on the residual oil in the micromodel. The size of the area is 6 × 6 mm. (**a**) water; (**b**) 0.1 wt% SiO_2_; (**c**) 0.25 wt% SiO_2_; (**d**) 0.5 wt% SiO_2_; (**e**) 1 wt% SiO_2_; (**f**) 2 wt% SiO_2_.

The analysis of the final distribution patterns of the oil fraction in the micromodel showed a significant difference obtained when displacing with water and suspensions (Figure 9). A significant residual oil saturation remains in the washed-up zone during displacement by water. Here, the oil remains in the form of large areas filled with oil, which is retained by capillary forces. Using SiO_2_ suspensions reduces the residual oil saturation. Figure 10 shows the enlarged areas of the micromodel (see the blue frame in Figure 9) with a size of 6 × 6 mm^2^, demonstrating the effect of the nanoparticle concentration on the residual fraction of oil in the micromodel. As a result of the process of displacement by SiO_2_ suspensions, the size of large areas, filled with oil, decreases. At that, this effect strengthens with an increase in the concentration of SiO_2_ nanoparticles in the suspension.

**Figure 11:**
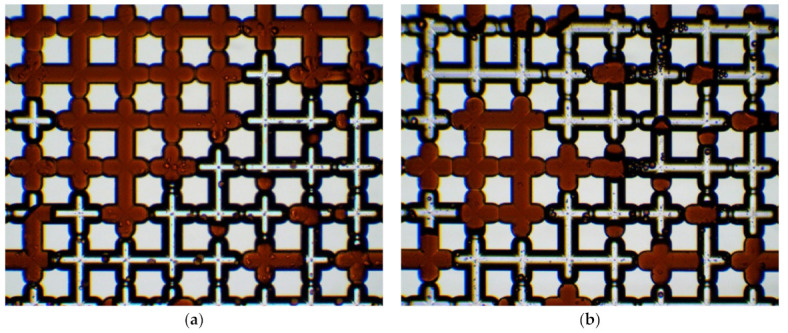
Photos of residual oil in a micromodel when flooding with water (**a**) and 2 wt% SiO_2_ suspension (**b**), obtained under a microscope.

Microscopy allowed it to be revealed that the main factor affecting the proportion of residual oil is the surface wettability (Figure 11). The addition of nanoparticles in distilled water reduces the oil wettability of the surface and changes the interfacial tension forces. With an increase in the concentration of nanoparticles, oil remains in the form of individual droplets in smaller pores. All this affects the reduction of residual oil in a microporous medium when flooding with suspensions (Figure 11).

**Figure 12:**
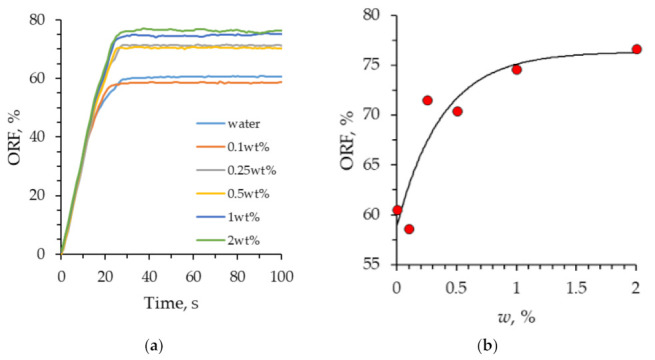
(**a**) The dynamic pattern of the oil recovery factor in the process of oil displacement from the microfluidic chip; (**b**) The dependence of the oil recovery factor on the concentration of nanoparticles.

An analysis of the time-dependent dynamic pattern of the oil recovery factor gave the following results (Figure 12). At the initial time point, the oil recovery factor increases linearly (in proportion to the displacing fluid flow rate). After the displacing fluid reaches the exit from the microporous medium, the growth of the oil recovery factor slows down. After 25 s, the displacement process becomes stationary, and further pumping of the displacing fluid practically does not change the oil recovery factor. Figure 12b shows the dependence of the final oil recovery factor on the concentration of SiO_2_ nanoparticles in the suspension. It was revealed that, when flooded with water, the oil recovery factor was 60%. As the concentration of nanoparticles increases, the oil recovery factor increases. When flooding with 2 wt% SiO_2_ suspension, the oil recovery factor reached 76%. At that, it should be noted that a significant effect can already be reached, even at small concentrations of nanoparticles (above 0.25 wt%).

## 4. Conclusions

A series of experiments using a microfluidic porous chip was conducted to study the process of flooding with SiO_2_ nanosuspensions. The dynamic pattern of the oil displacement process by water and SiO_2_ suspension depending on time was obtained. As a result of studying the pressure drop in the microfluidic chip, it was found that SiO_2_ nanoparticles affect the dynamic pattern of pressure drop during flooding. This is due to an increase in the breakthrough time of suspension from porous medium.

The effect of the concentration of nanoparticles on increasing the oil recovery factor has been investigated. The oil recovery factor during flooding with SiO_2_ suspension with a low concentration of nanoparticles was close to water (ORF = 60%). With an increase in the concentration of nanoparticles, an increase in the coefficient of oil recovery was observed, and its establishment on a plateau (ORF = 76%) at high concentrations.

Many factors are affecting the oil recovery factor. These are viscosity, density, interfacial tension, and wettability [39]. However, microscopy allowed us to reveal that the main factor affecting the oil recovery factor is surface wettability. The addition of nanoparticles to distilled water reduces the oil wettability of the surface and changes the interfacial tension forces, contributing to the washout of capillary-retained oil [40]. Microfluidic research can be extremely useful for understanding the oil displacement process mechanisms. The pressure drop in the displacement process was also measured. It is shown that, when oil is displaced from the microfluidic chip, the pressure losses weakly depend on the concentration of nanoparticles. This is very important for the practical application of nanoparticles.

## Figures and Tables

**Table 1:** Parameters of a porous microfluidic chip.

Parameter	Value
microchannel cross-section	100 × 110 µm
pore cross-section	Ø85 µm and Ø63 µm
input channel length, including bifurcations	27.7 mm
output channel length, including bifurcations	99.2 mm
the total length of the porous channel	4800 mm
inlet channel volume	0.9 µL
outlet channel volume	3.2 µL
porous space volume	38 µL
surface roughness of channels	5 nm
channel coating	hydrophilic

**Table 2:** Density and viscosity of the fluids used at 25 °C.

Fluid	Density, g cm^−3^	Viscosity, mPa s
oil	0.8510	24.6
distilled water	0.9970	0.890
0.1 wt% SiO_2_	0.9975	0.892
0.25 wt% SiO_2_	0.9984	0.896
0.5 wt% SiO_2_	0.9997	0.902
1 wt% SiO_2_	1.003	0.914
2 wt% SiO_2_	1.008	0.939
